# Peripheral axonal excitability in hemiplegia related to subacute stroke

**DOI:** 10.3906/sag-2004-306

**Published:** 2020-12-17

**Authors:** Zeynep TURAN, Murat ZİNNUROĞLU

**Affiliations:** 1 Department of Physical Medicine and Rehabilitation, Koç University Hospital, İstanbul Turkey; 2 Department of Physical Medicine and Rehabilitation, Faculty of Medicine, Gazi University, Ankara Turkey

**Keywords:** Stroke, peripheral axonal excitability, hemiplegia, membrane hyperpolarization

## Abstract

**Background/aim:**

This study aims to investigate peripheral nerve excitability in patients with subacute stroke.

**Materials and methods:**

The study was performed in 29 stroke patients within the subacute period and 29 healthy controls using QTRAC software and TRONDNF protocol. The threshold electrotonus, recovery cycle, stimulus-response, strength-duration, and current-threshold relationships were recorded.

**Results:**

The membrane was more hyperpolarized, and excitability was decreased in the hemiplegic side. The impairment of inward rectifying channel function, degree of hyperpolarization, and decrease of excitability were directly related to the Brunnstrom stages, which were more pronounced in lower stages.

**Conclusion:**

The lower motor neurons were affected at the level of axonal channels as a result of upper motor neuron lesions. It can be due to dying back neuropathy, homeostasis, and neurovascular regulation changes in the axonal environment, activity-dependent plastic changes, loss of drive coming from the central nervous system, or a combination of these factors.

## 1. Introduction

Hemiplegia is a unilateral neuromuscular dysfunction resulting from cerebral pathologies like stroke [1]. The existence of atrophy that is not expected with an upper motor lesion following a cerebral lesion has caused an investigation of possible lower motor neuron pathologies in these patients [2,3]. Physiological changes such as motor unit loss, denervation potentials in plegic muscles, and morphological changes have been reported in various morphological and electrophysiological studies [2,4]. Contradictory results have been obtained regarding nerve conduction in the hemiplegic extremity compared to the normal extremity in nerve conduction studies. It was concluded that lower motor neurons were also affected following an upper motor neuron lesion [5–7].

Conventional electrophysiological tests used for nerve functions focus on the number of fibers providing conduction and their conduction rates. However, this provides limited information about the axonal membrane. The threshold tracking (TT) technique tests axonal excitability (AE) based on axonal membrane characteristics in the stimulus zone. These methods are sensitive to the membrane potential and its changes which are caused by activation of ion channels and electrogenic ion pumps [7–9]. In short, AE measurement provides information about the membrane potential and biophysical features of axons. The number of nerve excitability studies in neuromuscular disorders has been increasing by using a semiautomatic QTRAC program that was written and developed by Bostock and enables nerve excitability tests to be conducted within 10–15 min [8,10]. This technique tests the features of nerve and axonal membrane at the point of stimulation [10]. The TT method is not used in daily practice yet; however, it provides some unique information about nerve function [9].

Nerve conduction velocity has not been found to change in the affected side of stroke patients in some studies, but was found to slow down in others. Contemporarily, excitability studies are the best method to obtain information on whether a peripheral axonal effect is present. This study aimed to investigate peripheral AE in cerebral stroke patients within the subacute period that had not been investigated previously.

## 2. Materials and methods

Thirty-eight hemiplegic patients during the subacute period and 39 control subjects from the inpatient clinic were enrolled in the study. Approval from the Medical Ethics Committee was obtained before the study. All subjects read and signed the consent form. The study was supported by the Gazi University Scientific Research Projects Unit.

Both affected and unaffected upper extremities of subacute stroke patients with ischemic brain injury and a single upper extremity of control subjects were evaluated. The inclusion criteria were as follows: aged between 18 and 75 years, volunteered to participate in the study, had a negative peripheral neuropathy history, had a diagnosis of stroke according to the World Health Organization for the stroke group, and had a negative stroke history for the control group. Exclusion criteria were as follows: presence of peripheral neuropathy or cervical radiculopathy, unwilling to participate in the study, unable to continue participation due to device-related problems during the study, and unable to provide an appropriate position of the extremity during the study due to reasons like excessive spasticity.

Age, sex, date of stroke, affected half of the body, Brunnstrom stage, neurological examination results, history, and physical examination findings of the patients were recorded. The presence of peripheral neuropathy was excluded by nerve conduction study at the baseline examination. An AE study was applied to both the affected and unaffected upper extremities of the hemiplegic patients and a single upper extremity of the control group by targeting motor axons. The electromyography signal was amplified by a Neuromatic electromyography device with analog output and then digitized by a National Instruments analog-to-digital converter data card. Stimulus waveforms generated by the computer were converted into currents by the Digitimer DS5 isolated linear bipolar constant current stimulator (output +/– 50 mA).

The stimulus currents were administered by nonpolarized Ag-AgCl inner surface electrodes by placing the active electrode on the median nerve on the wrist and the reference electrode 10 cm proximal and slightly radially on the muscle. The compound muscle action potential was recorded from the abductor pollicis brevis muscle using superficial electrodes with the active electrode on the motor point of the muscle and the reference electrode on the proximal phalanx.

Stimulation and recording were controlled with the QTRAC (Copyright Institute of Neurology, London, UK) software by using the TRONDNF protocol with the IV last option. The stimulus-response relationship, strength-duration relationship, threshold electrotonus (TE), recovery cycle, and current-threshold relationship were recorded in order. The test lasted about 10 min. The skin temperature of the workplace was monitored throughout the examination, making sure it did not fall under 32 °C.

Excitability studies on sensory axons in the median nerve could not be performed due to environmental artefacts and device-related problems.

The automatic data analysis program included in the QTRAC (London, UK) database was used for statistical analyses. Using the Lilliefors test, the program automatically calculated whether the data were distributed normally. The 2-sample t-test was used to compare parametric variables between two groups and the Mann–Whitney U test was run for nonparametric variables. The variables were identified as mean ± SD or median (minimum–maximum) and the statistical significance level was accepted as P < 0.05.

## 3. Results

This study was conducted at the Motor Control Laboratory of the Physical Medicine and Rehabilitation and Neurology Departments of the Gazi University Faculty of Medicine. The study evaluated 77 subjects. Excluded were 9 patients from the study group and 10 from the control group due to peripheral neuropathy, difficulty in giving the appropriate position due to spasticity, being unwilling to participate in the study because of pain or device-related, technical, or other reasons. The study was completed with 29 patients in the stroke group and 29 patients in the control group.

The mean age and sex of the patients in both groups were similar (P = 0.057 and P = 0.21, respectively). The mean age was 59.38 years in the stroke group and 53.28 in the control group.

The hand motor stage in the stroke patients according to the Brunnstrom staging system was 1–3 in 12 patients and 4–6 in 17 patients.

### 3.1. Comparison of the stroke-affected extremity group and the control group

The peak response, the TEd20 (10–20 ms) value in the stroke-affected extremity group was statistically significantly lower than the control group. No statistically significant difference was found in rheobase and strength-duration time constant (τSD) values and the current-threshold relationship. The relative refractory period (RRP) and refractoriness at 2 and 2.5 ms were significantly higher and late subexcitability was significantly lower in the stroke-affected extremity group (Table 1).

**Table 1:** Significant parameters in comparison of the stroke-affected extremity group and the control group.

	Mean ± SD (number) / Median (min-max)		P-value
	Stroke-affected extremity	Control group	
Peak response (mV)	2.864 (0.091-7.054) (26)	4.117 (0.595-5.824) (29)	P = 0.000**
RRP (ms)	3.148 ± 1.03 (29)	2.737 ± 1.02 (29)	P = 0.000**
Subexcitability (%)	11.25 ± 0.753 (29)	13.68 ± 0.804 (29)	P = 0.029*
Refractoriness (2.5 ms)	20.85 ± 4.05 (23)	10.12 ± 2.54 (29)	P = 0.022*
Refractoriness (2 ms)	56.82 (20.75-150.3) (17)	42.23 (12.4 -103.3) (29)	P = 0.035*
TEd20 (10–20 ms)	37.18 ± 0.676 (29)	39.06 ± 0.636 (29)	P = 0.045*

*: P < 0.05; **: P < 0.01 SD: Standard deviation; RRP: Relative refractory period; TE: Threshold electrotonus; mV: millivolt; ms: millisecond

### 3.2. Comparison of the Brunnstrom stage 1st, 2nd, 3rd group and the control group (Figure 1)

**Figure 1:**
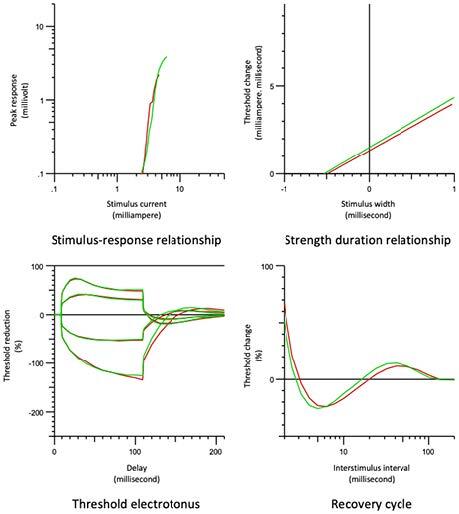
Comparison of the stroke-affected extremity group (red line) and the control group (green line).

The peak response, superexcitability (5 ms), late subexcitability, and minimum and resting current-threshold curve slope in the Brunnstrom stage 1st, 2nd, 3rd group were significantly lower than the control group. The stimulus-response curve slope, the TEh (90–100 ms) value, RRP, and refractoriness at 2 ms were statistically significantly higher in the stroke group. No statistically significant difference was found in the rheobase and τSD values between the groups (Table 2).

**Table 2:** Significant parameters in comparison of the Brunnstrom stage 1st, 2nd, 3rd group and the control group.

	Mean ± SD (number) / Median (min-max)		P-value
	Brunnstrom 1st, 2nd, 3rd group	Control group	
Stimulus- response curve	6.196 ± 1.05 (11)	4.235 ± 1.09 (29)	P = 0.011*
Peak response (mV)	2.872 (0.74–4.93) (11)	4.117 (0.595–5.824) (29)	P = 0.000**
Resting I/V curve	0.4889 ± 0.0301 (12)	0.5556 ± 0.0165 (27)	P = 0.040*
Minimum I/V curve	0.1898 ± 0.0142 (12)	0.2441 ± 0.0146 (28)	P = 0.029*
RRP (ms)	3.323 ± 1.06 (12)	2.737 ± 1.02 (29)	P = 0.000**
TEh (90 - 100 ms)	-147.5 ± 9.53 (12)	-125.5 ± 4.06 (29)	P = 0.015*
Subexcitability (%)	10.5 ± 1.13 (12)	13.68 ± 0.804 (29)	P = 0.032*
Refractoriness (2 ms)	56.82 (46.76–150.3) (5)	42.23 (12.4–103.3) (29)	p = 0.033*
Superexcitabity (5 ms)	-20.58 ± 2.53 (12)	-25.68 ± 1.09 (29)	P = 0.032*

*: P < 0.05; **: P < 0.01 SD: Standard deviation; RRP: Relative refractory period; TE: Threshold electrotonus; I/V: Current-voltage; mV: millivolt; ms: millisecond

### 3.3. Comparison of the Brunnstrom stage 4th, 5th, 6th group and the control group

Peak response in the Brunnstrom stage 4th, 5th, 6th group was significantly lower than the control group. TEh (20–40 ms), TEd20 (10–20 ms), RRP, and refractoriness values at 2.5 ms were significantly higher in the stroke group. No statistically significant difference was found in the rheobase and τSD values and current-threshold relationship between the groups (Table 3).

**Table 3:** Significant parameters in comparison of the Brunnstrom stage 4th, 5th, 6th groups and the control group.

	Mean ± SD (number) / Median (min - max)		P-value
	Brunnstrom 4th, 5th, 6th group	Control group	
Peak response (mV)	2.783 (0.091–7.054) (15)	4.117 (0.595–5.824) (29)	P = 0.000**
RRP (ms)	3.031 ± 1.04 (17)	2.737 ± 1.02 (29)	P = 0.006**
Refractoriness (2.5 ms)	22.79 ± 4.99 (16)	10.12 ± 2.54 (29)	P = 0.014*
TEh (20-40 ms)	-93.59 ± 2.39 (17)	-100.3 ± 2.02 (29)	P = 0.041*
TEd20 (10–20 ms)	35.64 ± 0.743 (17)	39.06 ± 0.636 (29)	P = 0.001**

*: P < 0.05; **: P < 0.01 SD: Standard deviation; RRP: Relative refractory period; TE: Threshold electrotonus; mV: millivolt; ms: millisecond

### 3.4. Comparison of the Brunnstrom stage 1st, 2nd, 3rd group and the Brunnstrom stage 4th, 5th, 6th group

No statistically significant difference was found between the Brunnstrom stage 1st, 2nd, 3rd group and Brunnstrom stage 4th, 5th, 6th group for stimulus-response curve, rheobase, and τSD, recovery cycle values and the current-threshold relationship. TEd20 (10–20 ms), TEd20 (peak) and TEh (90–100 ms) values were statistically significantly higher in the Brunnstrom stage 4tht, 5th, 6th group (P = 0.004, P = 0.037, and P = 0.006, respectively).

### 3.5. Comparison of the stroke-affected extremity group and the unaffected extremity group (Figure 2)

No statistically significant difference was found between the stroke-affected extremity and the unaffected group for stimulus-response curve, rheobase, and τSD, TE, and recovery cycle values. The resting current-threshold curve slope was statistically significantly lower in the affected group (P = 0.01975).

**Figure 2:**
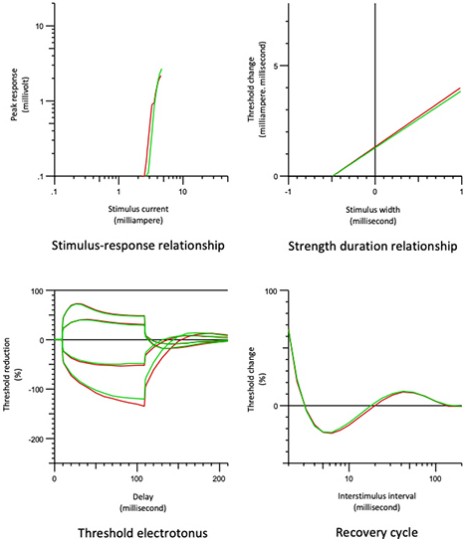
Comparison of the stroke-affected extremity group (red line) and the unaffected extremity group (green line).

### 3.6. The comparison between the stroke-unaffected group and the control group (Figure 3)

Peak response, TEh20 (10–20 ms), and TEh (10–20 ms) values in the stroke-unaffected extremity group were significantly lower and the stimulus-response curve slope and RRP were significantly higher than the control group. No statistically significant difference was found for rheobase, τSD values, and the current-threshold relationship between the groups (Table 4).

**Figure 3:**
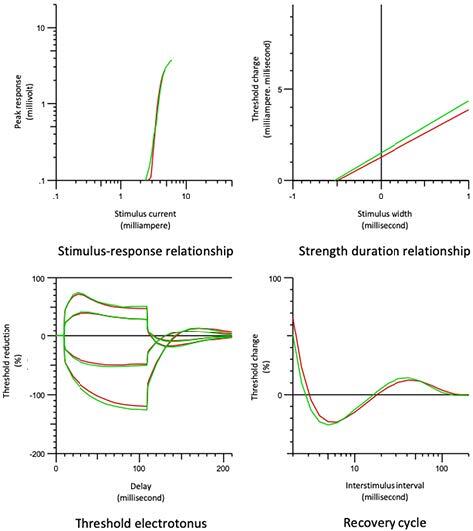
Comparison of the stroke-unaffected group (red line) and the control group (green line).

**Table 4:** Significant parameters in comparison of the stroke-unaffected group and the control group.

	Mean ± SD (number) / Median (min-max)		P-value
	Stroke-unaffected extremity group	Control group	
Stimulus-response curve	5.738 ± 1.06 (29)	4.235 ± 1.09 (29)	P = 0.005**
Peak response (mV)	3.3 (0.041 - 4.957) (29)	4.117 (0.595 - 5.824) (29)	P = 0.000**
RRP (ms)	3.028 ± 1.03 (29)	2.737 ± 1.02 (29)	P = 0.011*
TEh (10–20 ms)	-76.31 ± 1.94 (29)	-81.07 ± 1.3 (29)	P = 0.044*
TEh20 (10–20 ms)	-37.98 ± 1.03 (29)	-41.02 ± 0.657 (29)	P = 0.014*

*: P < 0.05; **: P < 0.01 SD: Standard deviation; RRP: Relative refractory period; TE: Threshold electrotonus; mV: millivolt; ms: millisecond

## 4. Discussion

The peak response was lower and latency was prolonged in the affected extremity group compared to the control group, while the stimulus-response curve slope was nearly significantly increased (P = 0.05). Hyperpolarization causes an increase in the stimulus-response curve slope [11]. The TEd20 (10–20 ms) in TE when depolarizing current spreads from the node to the internodal region [12] is decreased; this decrease in stroke patients indicates reduced depolarization, and thus reduced excitability. The RRP was prolonged, the refractoriness at 2 and 2.5 ms was increased, and subexcitability was decreased in the recovery cycle. The increase in membrane hyperpolarization in stroke results in the opening of more Na+ channels (increased activation) during the development of the action potential [12] and thus prolongation of the absolute refractory period. This period causes reactive depolarization and cannot be measured by the TT method. The more Na+ channels are opened, the more Na+ channels will be closed during recovery, which prolongs the RRP. This indicates decreasing excitability [13]. In a stroke, the membrane is more hyperpolarized, meaning an excessive (-) charge within the cell, leading to a reduction in the activation of K+ channels where ion flows from the high gradient inside to the low gradient outside and accordingly, there is a decrease in late subexcitability [13]. In conclusion, the membrane in the affected extremity of stroke patients was more hyperpolarized and excitability was decreased compared to the control group.

The latency prolonged, the peak response decreased, and the stimulus-response curve slope increased in the Brunnstrom stage 1st, 2nd, 3rd group compared to the control group. This increase, which was the most prominent in lower stage patients, corresponds to the increase in hyperpolarization that was higher in the lower stages. The superexcitability period [10,12,13], when the axon is stimulated more easily with the decrease of the threshold current power required for 10–30 ms following the refractory period, decreases the following depolarization [10,13] and increases the following hyperpolarization [10,13]. The depolarization of the Ranvier node stimulates adjacent internodes and leaves the current under the myelin sheath along the low resistance path after a short time. This creates a negative after-potential recorded extracellularly or a depolarizing after-potential (dap) recorded intracellularly. The weaker currents required to overcome membrane depolarization produce smaller dap and less supernormality [14]. The dap is decreased as a result of the reactive depolarization occurring in the membrane in stroke and this decreases superexcitability, resulting in decreased excitability. Parameters indicating the inward rectifier channel (IRC) function in TT are the S3 phase in TE and the hyperpolarization phase in the current- threshold slope. The increase in TEh (90–100 ms) [13] and the decrease in the hyperpolarization phase curve in the current-threshold slope [8] seen in low-stage acute stroke patients show the decrease in the function or expression of the IRC [15]. IRC becomes activated by membrane hyperpolarization and flow of positive ion, mainly K+, towards inside, unlike the concentration gradient, thus depolarizes the membrane potential and reverse the low membrane potential [10,13]. These channels play a role in the regulation of the axonal membrane potential and excitability [10,16]. They restrict hyperpolarization and the decrease in excitability throughout the prolonged nerve stimulation. According to the results of this study, the main problem in stroke is the IRC that is activated with hyperpolarization. When these channels do not function properly, membrane hyperpolarization cannot be restricted, and membranes remain hyperpolarized with decreased excitability. As a result, IRC function or expression was found to be decreased in low-stage stroke patients compared to the control group with the membrane remaining more hyperpolarized and the excitability decreased.

TEd20 (10–20 ms), TEh (20 - 40), and TEh (90–100) in TE were increased and the resting current-threshold curve slope decreased in Brunnstrom 1st, 2nd, 3rd group compared to the Brunnstrom 4th, 5th, 6th group. The parameters indicating IRC function at TT, as mentioned previously, are the S3 phase at TE and the hyperpolarization phase at the current-threshold slope. The change in these parameters in low-stage patients shows that the function of this channel activated by hyperpolarization is decreased. When we evaluate the curve sections formed by the depolarizing and hyperpolarizing current as a whole in TE, the low-stage patients show a fanning out appearance compared to high-stage in a similar way seen in a severe group of cerebellar infarct patients [16]. The membrane became hyperpolarized due to the decrease in IRC function in lower stages, and the membrane resistance has increased in already hyperpolarized axons. This might be due to the opening of fewer K+ channels, which leads to a bigger change in the amplitude of the response obtained by the conditioned constant current and it was observed in the form of fanning out [13,16]. Another condition that causes a fanning out appearance is compensatory hyperactivity of the Na+-K+-ATPase pump caused by a postischemic state with the resultant membrane hyperpolarization [13]. As a result, as the stage decreases, the IRC function decreases, the excitability decreases, and the membrane hyperpolarization increases.

The resting current-threshold curve slope decreased and the TEh (90–100 ms) increased close to the statistical significance level in the affected extremity group compared to the unaffected extremity group. This shows that the IRC expression activated by hyperpolarization decreases compared to the unaffected extremity group. This is similar to the decrease in the stroke group and low-stage stroke group as compared to the control group.

The peak response decreased and the stimulus-response curve slope increased in the unaffected extremity group compared to the control group. The RRP was prolonged in the recovery cycle. TEh20 (10–20 ms) and TEh (10–20 ms) were decreased in TE. When the curve sections formed by the depolarizing and hyperpolarizing current in TE were evaluated as a whole, the stroke patients had a relative fanning-in appearance on the unaffected side compared to the control group. The membrane remained depolarized due to an increase in IRC function on the unaffected side and the membrane resistance decreased in this predepolarized axon as more K+ channels were open. This caused a smaller change in the response amplitude to the conditioned constant current and it was observed as a fanning-in appearance [13]. Another condition that causes a fanning-in appearance is ischemia, which paralyzes the oxygen-dependent Na+-K+-ATPase pump in axon and leads to membrane depolarization [13]. Changes in the stimulus-response curve and RRP may be developed due to the compensation of this depolarization. In conclusion, the membrane became more depolarized and excitability increased in the stroke-unaffected extremity group compared to the control group.

While nerve conduction velocity slowing in the hemiplegic extremity has been reported in some studies [3,5,6,17], while no significant difference was reported in others [2]. This slowing down was attributed to the trophic effect loss from the upper center by Samusik [5]. Takabe et al. believed that this velocity reduction could be due to low temperature in the extremity and used a correction of 2.0 m/s for each degree, but still found a significant slowing down and attributed this to atrophic thinning of the fibers [17]. They demonstrated the slowing of nerve conduction in the unaffected side, but less than the affected side [17]. They attributed this to degeneration occurring in the lower motor neurons after the upper motor neuron lesion. This excitability study revealed that lower motor neurons were affected, possibly due to the loss of trophic effect from the upper center, as suggested by Samusik [5].

In some studies, compound muscle action potential (CMAP) amplitudes on the plegic side have been reported to be significantly reduced compared to the healthy side and interpreted as an indicator of motor unit loss [2,4,18,19]. McComas et al. attributed this condition to an approximate 50% decrease in the motor unit number that can depolarize the muscle fiber in the hemiplegic muscle [4]. Kingery et al. reported that the CMAP amplitude decrement may reflect motor axon destruction, because the decrease in CMAP amplitude is correlated with the degree of spontaneous activities, and spontaneous activities result from motor axonal degeneration [20]. In this study, we found a significant decrease in peak response in both the affected and unaffected extremity compared to the control group, but no differences between the 2 extremities. As a result of the upper motor neuron lesion, synaptic input to the anterior horn cells decreases and this leads to functional inactivity [21], axon current deterioration, functionally active motor unit number decrement, and consequently a reduction in CMAP [22].

Takebe et al. required higher current intensity and longer stimulus duration for nerve stimulation in the plegic extremity and interpreted this as hypoexcitability. They thought that the decrease in excitability was due to a change in motor neurons [17]. They deduced this conclusion with routine nerve conduction studies, while our results supporting hypoexcitability on the affected side were obtained with the AE method.

The reason why the lower motor neuron is affected by an upper motor neuron lesion and peripheral nerve function changes in both affected and unaffected side could be explained in several ways. The primary sensorimotor cortex undergoes functional organization after peripheral nerve injury [22]. In animal studies, it has been shown that cortical plasticity occurs within hours [22]. Since there is such a strong relationship between the central and peripheral nervous system as suggested by Van Kaujik et al., an upper motor neuron lesion may also cause a functional change in the lower motor neuron [22]. The cortical lesion distorts the input to the affected spinal motor neuron, supraspinal control is impaired, and the motor neuron is affected [18]. Changes in motor neuron properties affect the ion channels and pumps and may alter AE.

We thought that the reason for the changes in this study could be “dying back neuropathy”. This concept was first revealed by McComas [21]. After an upper motor neuron lesion, some changes develop in the lower motor neuron; synaptic input and activation loss, functional inactivity and transsynaptic degeneration [21], axonal current impairment, and axonal degeneration, followed by neuromuscular transmission dysfunction in the motor endplate and a decrease in the number of functionally active motor units, namely a motor unit that can be stimulated by electrical stimulation, finally resulting in decreased CMAP amplitude [22]. The motor unit loss is neither prolonged nor recovered [22]. A mild enlargement can be seen the active motor units in the chronic phase of stroke, leading to the restoration of axonal function, collateral innervation, and an increase in the number of muscle fibers activated. CMAP, therefore, shows recovery in the chronic period [22]. In this study, we detected CMAP amplitude decrement in the acute-subacute period. The reason could be dying-back neuropathy.

Prolonged changes in impulse traffic can cause a long-term change in the behavior of the neurons and induce plasticity in cerebellar stroke [16]. This suggests that plasticity may play a role in the described abnormalities. As Jankelowitz et al. suggested, considering the lower IRC expression in the affected side and higher in the unaffected side in stroke, the channel alteration activated by hyperpolarization can present activity-dependent plastic change, as the unaffected side can be more used due to the deficit on the affected side [18]. Plasticity may be induced even in the subacute period without requiring too much time in more severe cases.

Our peripheral motor AE study conducted in subacute stage stroke patients revealed that the membrane was more hyperpolarized and excitability decreased on the affected side compared to the unaffected side and the control group. IRC function on the affected side decreased compared to the unaffected side. The decrease in IRC function, excitability, and membrane hyperpolarization became more prominent in lower stages of stroke. The membrane was more depolarized and the excitability increased in the unaffected side of the stroke patients compared to the control group. The lower motor neurons are also affected at the level of axonal channels as a result of an upper motor lesion. These variations can be related to dying-back neuropathy, homeostasis, and neurovascular regulation changes occurring in the axon and its environment, activity-dependent plastic change, loss of the driving stimulus coming from the upper center, or the common result of all of these factors.

In future studies, a higher number of patients consisting of all Brunnstrom stages could be evaluated. Patients with spasticity could also be evaluated according to the Ashworth spasticity scale. Sensory axonal excitability studies could be performed in conjunction with microneurography to evaluate patients with and without the shoulder-hand syndrome.

## Informed Consent

The study protocol received institutional review board approval and that all participants provided informed consent in the format required by the Gazi University review board (2012/1330.

## Abbreviations

AE: Axonal excitability

CMAP: Compound muscle action potential

Dap: Depolarizing after-potential

IRC: Inward rectifier channels

RRP: Relative refractory period

τSD: Strength-duration time constant

TE: Threshold electrotonus

TT: Threshold tracking
